# Hypertrophic Rhinitis Treated Successfully with Adrenalin Chloride Solution

**Published:** 1901-06

**Authors:** Franklin W. Bock

**Affiliations:** Dansville, N. Y.


### HYPERTROPHIC RHINITIS TREATED SUCCESSFULLY WITH ADRENALIN CHLORIDE SOLUTION.

By FRANKLIN W. BOCK, M. D., Dansville, N. Y.

MISS —— came to me suffering from what, at first sight, appeared to be a very aggravated case of coryza; the nose was red and swollen, the nostrils excoriated, the eyes suffused with tears continually. The patient said that for several years she had not been able to breathe through the nose without great effort, and for the last four months she had suffered very much from an increase of the symptoms, being able to breathe through the nose but seldom.

Examination revealed an enormous hypertrophied condition of all the soft tissues of the nose, of a very spongy type, the probe being easily imbedded deeply in the turbinated bodies. I tried a number of remedies without any very good results. Cocaine was not used for good reasons, even to relieve the suffering.

I began the use of adrenalin by making two applications daily, all of the soft tissues of the nose being thoroughly contracted each time. The effect of the first application lasted about two hours. At the time of the second application the swelling did not seem to be quite so marked. I made two applications daily for three days, and then one a day for four days more. Each day the swelling seemed to be permanently less and at the end of the seventh day, the nose was in quite a normal condition; so I discontinued treatment with adrenalin, but kept up the use of ordinary cleansing applications. I had the case under treatment for ten days after the discontinuation of the adrenalin, and at that time the swelling had not returned.

I am also treating acute coryza with greater success than ever before. Everyone knows how hard it is to cleanse a nose which is swollen shut with a bad cold. I have not been able to do it very successfully without the use of cocaine—and that I will not use—until I began to use adrenalin. I now contract the tissues with adrenalin, and then give the nose a good thorough cleaning. The patient is relieved and cured quicker than by any treatment used heretofore.

